# Di­ethyl­ammonium di­hydrogen orthophosphate

**DOI:** 10.1107/S1600536814000464

**Published:** 2014-01-11

**Authors:** Peter Held

**Affiliations:** a Institut für Kristallographie, Universität zu Köln, Greinstrasse 6, D-50939 Köln, Germany

## Abstract

In the title molecular salt, [NH_2_(CH_2_CH_3_)_2_][H_2_PO_4_], two unique types of cations and anions, which are configurationally very similar, are present in the asymmetric unit. Both ions form sheets approximately parallel to (-1-1) linked by weak hydrogen bonds. The inter­connection within and between the sheets is reinforced by O—H⋯O and N—H⋯O hydrogen bonds involving the tetra­hedral H_2_PO_4_ anions and the ammonium groups.

## Related literature   

For preparative details, see: Hanna *et al.* (1999 ▶). For related structures, see: Averbuch-Pouchot *et al.* (1987 ▶); Held (2003 ▶). 
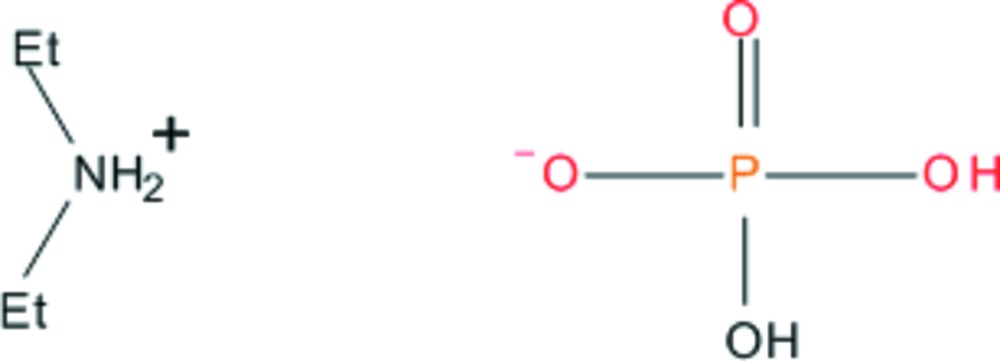


## Experimental   

### 

#### Crystal data  

C_4_H_12_N^+^·H_2_PO_4_^−^
*M* *_r_* = 171.13
Triclinic, 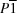

*a* = 8.3643 (6) Å
*b* = 8.8308 (15) Å
*c* = 11.6446 (12) Å
α = 88.219 (10)°
β = 83.649 (7)°
γ = 79.700 (7)°
*V* = 841.00 (18) Å^3^
*Z* = 4
Mo *K*α radiation
μ = 0.29 mm^−1^
*T* = 295 K
0.30 × 0.28 × 0.26 mm

#### Data collection  

Nonius MACH3 diffractometer
Absorption correction: ψ scan (North *et al.*, 1968 ▶) *T*_min_ = 0.858, *T*_max_ = 0.998
10831 measured reflections
5096 independent reflections
3164 reflections with *I* > 2σ(*I*)
*R*_int_ = 0.037
3 standard reflections every 100 reflections intensity decay: −6.3%

#### Refinement  

*R*[*F* ^2^ > 2σ(*F* ^2^)] = 0.040
*wR*(*F* ^2^) = 0.119
*S* = 0.98
5096 reflections
181 parameters
H-atom parameters constrained
Δρ_max_ = 0.37 e Å^−3^
Δρ_min_ = −0.39 e Å^−3^

### 

Data collection: *CAD-4* (Enraf–Nonius, 1989 ▶); cell refinement: *CAD-4*; data reduction: *WinGX* (Farrugia, 2012 ▶); program(s) used to solve structure: *SIR97* (Altomare *et al.*, 1999 ▶); program(s) used to refine structure: *SHELXL97* (Sheldrick, 2008 ▶); molecular graphics: *ATOMS* (Dowty, 2002 ▶) and *ORTEP-3 for Windows* (Farrugia, 2012 ▶)’; software used to prepare material for publication: *publCIF* (Westrip, 2010 ▶).

## Supplementary Material

Crystal structure: contains datablock(s) I, global. DOI: 10.1107/S1600536814000464/wm2794sup1.cif

Structure factors: contains datablock(s) I. DOI: 10.1107/S1600536814000464/wm2794Isup2.hkl

CCDC reference:

Additional supporting information:  crystallographic information; 3D view; checkCIF report

## Figures and Tables

**Table 1:** Hydrogen-bond geometry (Å, °)

*D*—H⋯*A*	*D*—H	H⋯*A*	*D*⋯*A*	*D*—H⋯*A*
O13—H13⋯O21^i^	0.82	1.78	2.5851 (19)	166
O14—H14⋯O12^ii^	0.82	1.83	2.6058 (19)	158
O24—H24⋯O22^iii^	0.82	1.95	2.585 (2)	133
O23—H23⋯O11^i^	0.82	1.84	2.620 (2)	158
N1—H1*A*⋯O22^iv^	0.90	1.88	2.779 (2)	174
N1—H1*B*⋯O21^v^	0.90	1.87	2.769 (2)	177
N2—H2*A*⋯O11^vi^	0.90	1.87	2.714 (2)	155
N2—H2*B*⋯O12	0.90	1.91	2.795 (2)	168

Symmetry codes: (i) 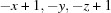; (ii) 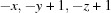; (iii) 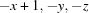; (iv) 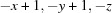; (v) 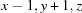; (vi) 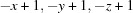.

**Fig. 1.**
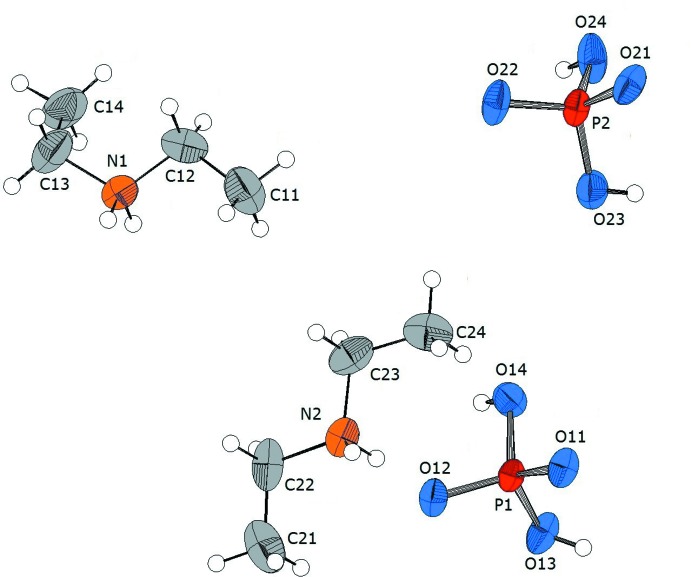
The molecular entities in the structure of [NH2(CH2CH3)2]+[H2PO4]-, showing the atom-numbering scheme. Displacement ellipsoids are drawn at the 50% probability level. H labels were omitted for clarity.

**Fig. 2.**
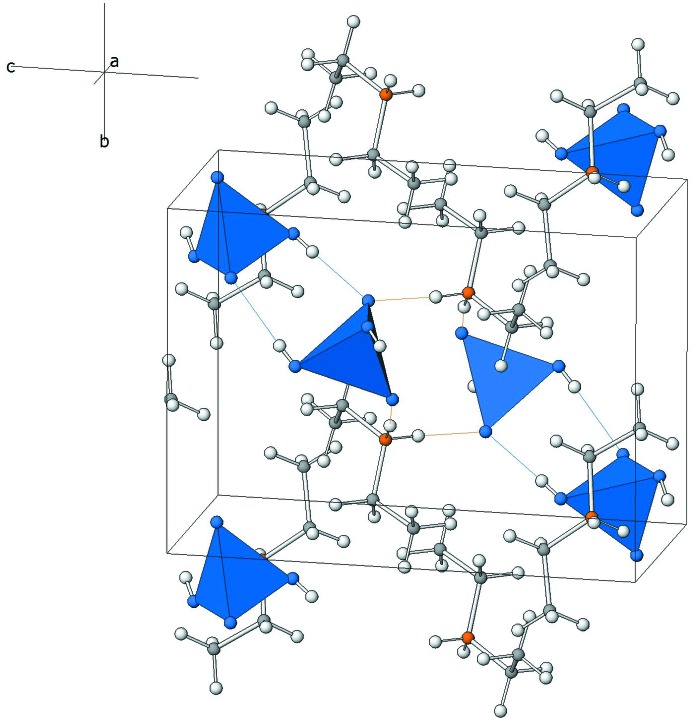
The unit cell of [NH2(CH2CH3)2]+[H2PO4]- with colour scheme: N (orange), O (blue), [H2PO4]-tetrahedra (blue), P (red), C grey) and H (white). Hydrogen bonds (light blue) between H2PO4 tetrahedra and hydrogen bonds (orange) between ammonium groups and H2PO4 tetrahedra are shown.

**Fig. 3.**
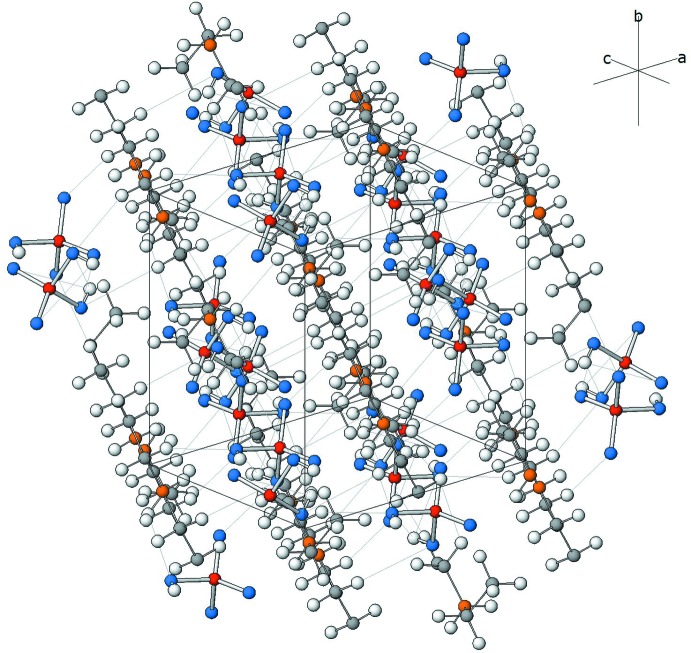
Clinographic projection of eight unit cells of [NH2(CH2CH3)2]+[H2PO4]-. The ionic units form sheets approximately parallel to (112). Hydrogen bonds (grey) interconnect the sheets.

## supplementary crystallographic information

### 1. Comment

In the course of a systematic search for new 'double salts' of simple secondary amines and monovalent cations of various inorganic acids (Averbuch-Pouchot *et al.*, 1987), the new structure of (C_2_N_2_H_10_)Li_2_(SO_4_)_2_ was described (Held, 2003). In continuation of these studies, sulfuric acid has been replaced with phosphoric acid in order to get an analogous lithium compound with a tetrahedral phosphoric unit. Moreover, ethylenediamine has been replaced with diethylamine. Surprisingly, lithium was not incorporated in the solid product and only the title compound, [NH_2_(CH_2_CH_3_)_2_]^+^[H_2_PO_4_]^-^, was finally obtained, the crystal structure of which is reported herein.

The crystal structure of [NH_2_(CH_2_CH_3_)_2_]^+^[H_2_PO_4_]^-^ consists of diethylammonium cations, NH_2_(CH_2_CH_3_)_2_^+^, and dihydrogen orthophosphate anions, H_2_PO_4_^-^ (Fig. 1). The ions form sheets approximately parallel to (112). The interconnection within and between the sheets is reinforced by a hydrogen bonding system between the tetrahedral dihydrogen orthophosphate groups on one hand and between the ammonium function and the H_2_PO_4_^-^ units on the other (Figs. 2,3; Table 1).

### 2. Experimental

The title compound was obtained by reaction of an aqueous solution of lithium dihydrogenposphate with diethylammine in a stoichiometric ratio 1:1 (Hanna *et al.*, 1999). The solution was kept at room temperature by cooling. The title compound crystallized by slow evaporation of the solvent at room temperature in form of colourless crystals with dimensions up to 4 mm within a few days.

Differential scanning calorimetry with a PerkinElmer DSC7 device in the temperature range from 183 K up to 293 K showed no significant feature.

### 3. Refinement

The H atoms were clearly discernible from difference Fourier maps. However, to all hydrogen atoms riding model contraints were applied in the least squares refinement, with C—H = 0.96 Å for methyl H atoms (*U*_iso_(H) = 1.5*U*_eq_(C)), with C—H = 0.97 (*U*_iso_(H) = 1.2*U*_eq_(C)) for methylene H atoms, with N—H = 0.90 Å (*U*_iso_(H) = 1.2*U*_eq_(N)) and with O—H = 0.82 Å (*U*_iso_(H) = 1.2*U*_eq_(O)).

### Crystal data

**Table:**

C_4_H_12_N^+^·H_2_PO_4_^−^	*Z* = 4
*M**_r_* = 171.13	*F*(000) = 368
Triclinic, *P*1	*D*_x_ = 1.352 Mg m^−^^3^
Hall symbol: -P 1	Mo *K*α radiation, λ = 0.71073 Å
*a* = 8.3643 (6) Å	Cell parameters from 25 reflections
*b* = 8.8308 (15) Å	θ = 21.0–26.0°
*c* = 11.6446 (12) Å	µ = 0.29 mm^−^^1^
α = 88.219 (10)°	*T* = 295 K
β = 83.649 (7)°	Parallelepiped, colourless
γ = 79.700 (7)°	0.30 × 0.28 × 0.26 mm
*V* = 841.00 (18) Å^3^	

### Data collection

**Table:**

Nonius MACH3 diffractometer	3164 reflections with *I* > 2σ(*I*)
Radiation source: fine-focus sealed tube	*R*_int_ = 0.037
Graphite monochromator	θ_max_ = 30.4°, θ_min_ = 2.5°
ω/2θ scans	*h* = −11→11
Absorption correction: ψ scan (North *et al.*, 1968)	*k* = −12→12
*T*_min_ = 0.858, *T*_max_ = 0.998	*l* = −16→16
10831 measured reflections	3 standard reflections every 100 reflections
5096 independent reflections	intensity decay: −6.3%

### Refinement

**Table:**

Refinement on *F*^2^	Primary atom site location: structure-invariant direct methods
Least-squares matrix: full	Secondary atom site location: difference Fourier map
*R*[*F*^2^ > 2σ(*F*^2^)] = 0.040	Hydrogen site location: inferred from neighbouring sites
*wR*(*F*^2^) = 0.119	H-atom parameters constrained
*S* = 0.98	*w* = 1/[σ^2^(*F*_o_^2^) + (0.0553*P*)^2^ + 0.1855*P*] where *P* = (*F*_o_^2^ + 2*F*_c_^2^)/3
5096 reflections	(Δ/σ)_max_ < 0.001
181 parameters	Δρ_max_ = 0.37 e Å^−^^3^
0 restraints	Δρ_min_ = −0.39 e Å^−^^3^

### Special details

**Table:**

Experimental. A suitable single-crystal was carefully selected under a polarizing microscope and mounted in a glass capillary.
Geometry. All e.s.d.'s (except the e.s.d. in the dihedral angle between two l.s. planes) are estimated using the full covariance matrix. The cell e.s.d.'s are taken into account individually in the estimation of e.s.d.'s in distances, angles and torsion angles; correlations between e.s.d.'s in cell parameters are only used when they are defined by crystal symmetry. An approximate (isotropic) treatment of cell e.s.d.'s is used for estimating e.s.d.'s involving l.s. planes.
Refinement. Refinement of *F*^2^ against ALL reflections. The weighted *R*-factor *wR* and goodness of fit *S* are based on *F*^2^, conventional *R*-factors *R* are based on *F*, with *F* set to zero for negative *F*^2^. The threshold expression of *F*^2^ > σ(*F*^2^) is used only for calculating *R*-factors(gt) *etc*. and is not relevant to the choice of reflections for refinement. *R*-factors based on *F*^2^ are statistically about twice as large as those based on *F*, and *R*- factors based on ALL data will be even larger.

### Fractional atomic coordinates and isotropic or equivalent isotropic displacement parameters (Å^2^)

**Table:**

	*x*	*y*	*z*	*U*_iso_*/*U*_eq_	
P1	0.16567 (5)	0.37327 (5)	0.60452 (4)	0.02736 (12)	
O11	0.33132 (15)	0.27994 (16)	0.61779 (12)	0.0362 (3)	
O12	0.16402 (16)	0.53915 (15)	0.56977 (12)	0.0365 (3)	
O13	0.05039 (16)	0.36883 (18)	0.71998 (12)	0.0426 (4)	
H13	0.0986	0.3119	0.7669	0.064*	
O14	0.08747 (18)	0.29249 (16)	0.51243 (13)	0.0418 (3)	
H14	−0.0034	0.3416	0.5037	0.063*	
P2	0.67897 (6)	−0.09624 (6)	0.11311 (4)	0.03332 (13)	
O21	0.83801 (17)	−0.20760 (18)	0.10779 (12)	0.0431 (4)	
O22	0.68455 (18)	0.04631 (17)	0.03943 (13)	0.0454 (4)	
O23	0.6256 (2)	−0.04343 (17)	0.24046 (13)	0.0502 (4)	
H23	0.6138	−0.1185	0.2816	0.075*	
O24	0.54703 (19)	−0.18437 (17)	0.07620 (15)	0.0540 (4)	
H24	0.4588	−0.1259	0.0784	0.081*	
C11	0.2499 (4)	0.5728 (3)	−0.0009 (3)	0.0663 (7)	
H11A	0.3010	0.4677	−0.0143	0.100*	
H11B	0.1790	0.5792	0.0702	0.100*	
H11C	0.3322	0.6346	0.0040	0.100*	
C12	0.1521 (3)	0.6303 (3)	−0.0985 (2)	0.0551 (6)	
H12A	0.2234	0.6223	−0.1705	0.066*	
H12B	0.0700	0.5668	−0.1043	0.066*	
N1	0.0712 (2)	0.7927 (2)	−0.08051 (14)	0.0389 (4)	
H1A	0.1462	0.8472	−0.0629	0.047*	
H1B	−0.0049	0.7962	−0.0192	0.047*	
C13	−0.0092 (4)	0.8696 (3)	−0.1815 (2)	0.0633 (7)	
H13A	−0.0518	0.9767	−0.1634	0.076*	
H13B	0.0719	0.8668	−0.2481	0.076*	
C14	−0.1460 (4)	0.7938 (4)	−0.2114 (2)	0.0674 (8)	
H14A	−0.1939	0.8467	−0.2759	0.101*	
H14B	−0.2275	0.7978	−0.1461	0.101*	
H14C	−0.1040	0.6884	−0.2311	0.101*	
C21	0.2697 (4)	0.9059 (3)	0.4832 (3)	0.0767 (9)	
H21A	0.2125	1.0088	0.4722	0.115*	
H21B	0.3793	0.9091	0.4988	0.115*	
H21C	0.2144	0.8581	0.5472	0.115*	
C22	0.2739 (3)	0.8160 (3)	0.3772 (2)	0.0530 (6)	
H22A	0.3283	0.8650	0.3122	0.064*	
H22B	0.1632	0.8138	0.3608	0.064*	
N2	0.36206 (19)	0.65586 (19)	0.39194 (14)	0.0387 (4)	
H2A	0.4643	0.6596	0.4076	0.046*	
H2B	0.3118	0.6126	0.4535	0.046*	
C23	0.3717 (3)	0.5556 (3)	0.2904 (2)	0.0524 (6)	
H23A	0.4293	0.5993	0.2242	0.063*	
H23B	0.2622	0.5520	0.2721	0.063*	
C24	0.4584 (3)	0.3952 (3)	0.3128 (2)	0.0599 (7)	
H24A	0.4627	0.3337	0.2456	0.090*	
H24B	0.4005	0.3509	0.3773	0.090*	
H24C	0.5675	0.3983	0.3297	0.090*	

### Atomic displacement parameters (Å^2^)

**Table:**

	*U*^11^	*U*^22^	*U*^33^	*U*^12^	*U*^13^	*U*^23^
P1	0.0192 (2)	0.0322 (2)	0.0307 (2)	−0.00441 (17)	−0.00520 (17)	0.00718 (18)
O11	0.0191 (6)	0.0458 (8)	0.0417 (8)	−0.0025 (5)	−0.0037 (5)	0.0122 (6)
O12	0.0325 (7)	0.0347 (7)	0.0448 (8)	−0.0102 (6)	−0.0119 (6)	0.0110 (6)
O13	0.0254 (7)	0.0588 (9)	0.0371 (7)	0.0040 (6)	0.0011 (6)	0.0160 (7)
O14	0.0397 (8)	0.0365 (7)	0.0507 (9)	−0.0017 (6)	−0.0193 (7)	−0.0035 (6)
P2	0.0281 (2)	0.0350 (3)	0.0388 (3)	−0.00980 (19)	−0.0092 (2)	0.0130 (2)
O21	0.0312 (7)	0.0590 (9)	0.0344 (7)	0.0011 (6)	−0.0025 (6)	0.0143 (7)
O22	0.0428 (8)	0.0464 (8)	0.0549 (9)	−0.0229 (7)	−0.0227 (7)	0.0253 (7)
O23	0.0586 (10)	0.0409 (8)	0.0447 (9)	0.0048 (7)	−0.0021 (7)	0.0069 (7)
O24	0.0470 (9)	0.0392 (8)	0.0850 (12)	−0.0202 (7)	−0.0334 (8)	0.0228 (8)
C11	0.0626 (17)	0.0470 (14)	0.092 (2)	−0.0091 (12)	−0.0194 (16)	−0.0012 (14)
C12	0.0502 (14)	0.0593 (15)	0.0571 (15)	−0.0138 (11)	0.0012 (11)	−0.0197 (12)
N1	0.0366 (9)	0.0481 (10)	0.0361 (9)	−0.0195 (8)	−0.0036 (7)	0.0022 (7)
C13	0.0762 (19)	0.0782 (18)	0.0452 (14)	−0.0365 (15)	−0.0179 (13)	0.0191 (13)
C14	0.0759 (19)	0.081 (2)	0.0537 (15)	−0.0226 (15)	−0.0304 (14)	0.0031 (14)
C21	0.074 (2)	0.0466 (15)	0.103 (2)	0.0002 (14)	0.0004 (18)	0.0028 (16)
C22	0.0306 (10)	0.0525 (13)	0.0738 (17)	−0.0069 (9)	−0.0045 (10)	0.0280 (12)
N2	0.0265 (8)	0.0485 (10)	0.0427 (9)	−0.0117 (7)	−0.0050 (7)	0.0104 (8)
C23	0.0418 (12)	0.0802 (18)	0.0401 (12)	−0.0225 (12)	−0.0078 (10)	0.0027 (12)
C24	0.0503 (14)	0.0718 (18)	0.0595 (16)	−0.0205 (13)	0.0058 (12)	−0.0170 (13)

### Geometric parameters (Å, º)

**Table:**

P1—O11	1.5013 (13)	C13—C14	1.501 (4)
P1—O12	1.5056 (14)	C13—H13A	0.9700
P1—O14	1.5673 (14)	C13—H13B	0.9700
P1—O13	1.5691 (14)	C14—H14A	0.9600
O13—H13	0.8200	C14—H14B	0.9600
O14—H14	0.8200	C14—H14C	0.9600
P2—O21	1.5027 (14)	C21—C22	1.482 (4)
P2—O22	1.5060 (14)	C21—H21A	0.9600
P2—O23	1.5613 (16)	C21—H21B	0.9600
P2—O24	1.5624 (15)	C21—H21C	0.9600
O23—H23	0.8200	C22—N2	1.487 (3)
O24—H24	0.8200	C22—H22A	0.9700
C11—C12	1.497 (4)	C22—H22B	0.9700
C11—H11A	0.9600	N2—C23	1.485 (3)
C11—H11B	0.9600	N2—H2A	0.9000
C11—H11C	0.9600	N2—H2B	0.9000
C12—N1	1.483 (3)	C23—C24	1.501 (4)
C12—H12A	0.9700	C23—H23A	0.9700
C12—H12B	0.9700	C23—H23B	0.9700
N1—C13	1.503 (3)	C24—H24A	0.9600
N1—H1A	0.9000	C24—H24B	0.9600
N1—H1B	0.9000	C24—H24C	0.9600
			
O11—P1—O12	115.38 (8)	N1—C13—H13B	109.1
O11—P1—O14	107.71 (8)	H13A—C13—H13B	107.9
O12—P1—O14	109.31 (8)	C13—C14—H14A	109.5
O11—P1—O13	110.08 (7)	C13—C14—H14B	109.5
O12—P1—O13	108.20 (8)	H14A—C14—H14B	109.5
O14—P1—O13	105.74 (9)	C13—C14—H14C	109.5
P1—O13—H13	109.5	H14A—C14—H14C	109.5
P1—O14—H14	109.5	H14B—C14—H14C	109.5
O21—P2—O22	114.38 (9)	C22—C21—H21A	109.5
O21—P2—O23	109.41 (8)	C22—C21—H21B	109.5
O22—P2—O23	107.50 (9)	H21A—C21—H21B	109.5
O21—P2—O24	107.55 (9)	C22—C21—H21C	109.5
O22—P2—O24	110.16 (8)	H21A—C21—H21C	109.5
O23—P2—O24	107.65 (10)	H21B—C21—H21C	109.5
P2—O23—H23	109.5	C21—C22—N2	110.6 (2)
P2—O24—H24	109.5	C21—C22—H22A	109.5
C12—C11—H11A	109.5	N2—C22—H22A	109.5
C12—C11—H11B	109.5	C21—C22—H22B	109.5
H11A—C11—H11B	109.5	N2—C22—H22B	109.5
C12—C11—H11C	109.5	H22A—C22—H22B	108.1
H11A—C11—H11C	109.5	C23—N2—C22	114.70 (18)
H11B—C11—H11C	109.5	C23—N2—H2A	108.6
N1—C12—C11	110.87 (19)	C22—N2—H2A	108.6
N1—C12—H12A	109.5	C23—N2—H2B	108.6
C11—C12—H12A	109.5	C22—N2—H2B	108.6
N1—C12—H12B	109.5	H2A—N2—H2B	107.6
C11—C12—H12B	109.5	N2—C23—C24	111.64 (19)
H12A—C12—H12B	108.1	N2—C23—H23A	109.3
C12—N1—C13	115.31 (19)	C24—C23—H23A	109.3
C12—N1—H1A	108.4	N2—C23—H23B	109.3
C13—N1—H1A	108.4	C24—C23—H23B	109.3
C12—N1—H1B	108.4	H23A—C23—H23B	108.0
C13—N1—H1B	108.4	C23—C24—H24A	109.5
H1A—N1—H1B	107.5	C23—C24—H24B	109.5
C14—C13—N1	112.3 (2)	H24A—C24—H24B	109.5
C14—C13—H13A	109.1	C23—C24—H24C	109.5
N1—C13—H13A	109.1	H24A—C24—H24C	109.5
C14—C13—H13B	109.1	H24B—C24—H24C	109.5

### Hydrogen-bond geometry (Å, º)

**Table:**

*D*—H···*A*	*D*—H	H···*A*	*D*···*A*	*D*—H···*A*
O13—H13···O21^i^	0.82	1.78	2.5851 (19)	166
O14—H14···O12^ii^	0.82	1.83	2.6058 (19)	158
O24—H24···O22^iii^	0.82	1.95	2.585 (2)	133
O23—H23···O11^i^	0.82	1.84	2.620 (2)	158
N1—H1*A*···O22^iv^	0.90	1.88	2.779 (2)	174
N1—H1*B*···O21^v^	0.90	1.87	2.769 (2)	177
N2—H2*A*···O11^vi^	0.90	1.87	2.714 (2)	155
N2—H2*B*···O12	0.90	1.91	2.795 (2)	168

Symmetry codes: (i) −*x*+1, −*y*, −*z*+1; (ii) −*x*, −*y*+1, −*z*+1; (iii) −*x*+1, −*y*, −*z*; (iv) −*x*+1, −*y*+1, −*z*; (v) *x*−1, *y*+1, *z*; (vi) −*x*+1, −*y*+1, −*z*+1.
